# Plant-growth regulators alter phytochemical constituents and pharmaceutical quality in Sweet potato (*Ipomoea batatas* L.)

**DOI:** 10.1186/s12906-016-1113-1

**Published:** 2016-05-28

**Authors:** Ali Ghasemzadeh, Daryush Talei, Hawa Z. E. Jaafar, Abdul Shukor Juraimi, Mahmud Tengku Muda Mohamed, Adam Puteh, Mohd Ridzwan A. Halim

**Affiliations:** Department of Crop Science, Faculty of Agriculture, Universiti Putra Malaysia, 43400 Serdang, Selangor Malaysia; Medicinal Plants Research Center, Shahed University, Tehran, Iran

**Keywords:** Plant-growth regulators, Sweet potato, Phenylalanine ammonia lyase, DPPH assay, β-carotene blenching assay, UHPLC

## Abstract

**Background:**

Sweet potato (*Ipomoea batatas* L.) is one of the most important consumed crops in many parts of the world because of its economic importance and content of health-promoting phytochemicals.

**Methods:**

With the sweet potato (*Ipomoea batatas* L.) as our model, we investigated the exogenous effects of three plant-growth regulators methyl jasmonate (MeJA), salicylic acid (SA), and abscisic acid (ABA) on major phytochemicals in relation to phenylalanine ammonia lyase (PAL) activity. Specifically, we investigated the total phenolic content (TPC), total flavonoid content (TFC), total anthocyanin content (TAC), and total β-carotene content (TCC). Individual phenolic and flavonoid compounds were identified using ultra-high performance liquid chromatography (UHPLC). Antioxidant activities of treated plants were evaluated using a 1,1-diphenyl-2-picrylhydrazyl (DPPH) assay and a β-carotene bleaching assay. Anticancer activity of extracts was evaluated against breast cancer cell lines (MCF-7 and MDA-MB-231) using MTT assay.

**Results:**

TPC, TFC, TAC, and TCC and antioxidant activities were substantially increased in MeJA-, SA-, and ABA-treated plants. Among the secondary metabolites identified in this study, MeJA application significantly induced production of quercetin, kaempferol, myricetin, gallic acid, chlorogenic acid, 3,5-dicaffeoylquinic acid, and 4,5-dicaffeoylquinic acid. Luteolin synthesis was significantly induced by SA application. Compared with control plants, MeJA-treated sweet potato exhibited the highest PAL activity, followed by SA and ABA treatment. The high DPPH activity was observed in MeJA followed by SA and ABA, with half-maximal inhibitory concentration (IC_50_) values of 2.40, 3.0, and 3.40 mg/mL compared with α-tocopherol (1.1 mg/mL). Additionally, MeJA-treated sweet potato showed the highest β-carotene bleaching activity, with an IC_50_ value of 2.90 mg/mL, followed by SA (3.30 mg/mL), ABA (3.70 mg/mL), and control plants (4.5 mg/mL). Extracts of sweet potato root treated with MeJA exhibited potent anticancer activity with IC_50_ of 0.66 and 0.62 mg/mL against MDA-MB-231 and MCF-7 cell lines respectively, compared to that of extracts of sweet potato treated with SA (MDA-MB-231 = 0.78 mg/mL; MCF-7 = 0.90 mg/mL) and ABA (MDA-MB-231 = 0.94 mg/mL; MCF-7 = 1.40 mg/mL). The results of correlation analysis showed that anthocyanins and flavooids are corresponding compounds in sweet potato root extracts for anticancer activity against breast cancer cell lines.

**Conclusions:**

MeJA has great potential to enhance the production of important health-promoting phytochemicals in sweet potato.

## Background

Sweet potato (*Ipomoea batatas* L.) is a dicotyledonous plant with tubers derived from swollen roots. It is widely consumed in many parts of the world and is considered a major crop [1]. The sweet potato has been widely studied both because of its economic importance and because it is associated with numerous health benefits [2]. The sweet potato contains phytochemicals with various pharmaceutical activities, which have antioxidant [3], anticancer activity [4], antidiabetic activity [5] and anti-inflammatory activity [6]. The pharmaceutical activity of the sweet potato is correlated with its phytochemical compounds. It has long been known that the sweet potato contains β-carotene, a precursor to vitamin A that helps prevent night blindness and other symptoms of vitamin A deficiency [7]. Besides carotenoids, sweet potatoes also contain bioactive compounds, including carotenoids, anthocyanins, phenolic acids, other flavonoids, and vitamin C [8]. Sweet potatoes also contain a unique blend of phenolic compounds, including hydroxycinnamic acids, which represent the primary phenolic antioxidant in most commercially available cultivars [9]. Among these phytochemicals, flavonoids and phenolics are particularly noted for having antioxidant and anticancer benefits because they exhibit strong superoxide-radical scavenging activity [10–12]. Thus, researchers are extremely interested in the effect of dietary phenolics. It is of particular interest whether phenol and flavonoid synthesis can be increased in crops through agronomic management techniques. Plants are able to integrate a wide variety of stimuli from both internal and environmental sources to alter their metabolic activities. Compounds known as elicitors can trigger the synthesis of phytochemicals, including phenolics and flavonoids, in fruits, vegetables, and herbs. Some major elicitors are jasmonic acid (MeJA), salicylic acid (SA), and abscisic acid (ABA) are known to be potent elicitors and defense phytohormones, and play significant roles as plant-growth regulators (PGRs) and regulators of plant defense responses against various biotic and abiotic stresses [13–15]. Phenolic and flavonoids synthesis is regulated by PGRs such as MeJA and SA [16–18]. Exogenous SA and ABA positively influence the levels of phytochemicals in ginger [16] and lettuces [19] respectively. Phenylalanine ammonia lyase (PAL) catalyzes the non-oxidative deamination of L-phenylalanine to trans-cinnamic acid and ammonia. This step directs carbon flow in the plant from the shikimate pathway to phenylpropanoid metabolism, a secondary metabolic pathway in higher plants and some other organisms that is mainly involved in defense mechanisms [20]. This pathway produces malonyl-CoA and p-coumaroyl-CoA, from which all phenols and flavonoids derive their carbon skeletons [21]. The first committed step for biosynthesis of the phenylpropanoid skeleton in higher plants is the deamination of L-phenylalanine to yield trans-cinnamic acid and ammonia. This reaction is catalyzed by phenylalanine ammonia-lyase (PAL) and is often regarded as a key step in the biosynthesis of the flavonoids [22]. Concomitant increases in the levels of PAL and flavonoid compounds have been demonstrated by previous studies [22, 23]. Accordingly, previous studies have demonstrated concomitant increases in PAL levels and phenolic or flavonoid compounds [24, 25]. Nakatani and Koda [26] demonstrated that jasmonic acid and its related compounds play an important physiological role in development of storage root. The result of recent study showed that foliar application of SA at concentration of 10^−5^M induced significantly antioxidant and anticancer activity of Malaysian young ginger varieties [16]. Also, exogenous ABA was found to stimulate anthocyanin and phenolics biosynthesis and increase antioxidant activity of muscadine grapes [27]. Poor nutrition is a major problem worldwide and there is considerable incentive to develop food-crops with improved nutritional content. Currently, it is important to gather relevant evidence on food-crops with high levels of potentially beneficial components. The high beneficial value of sweet potato renders it an excellent candidate for the investigation of how PGRs may alter phytochemical production. Specifically, we can test whether improving plant resistance to biotic and abiotic stresses through elicitor stimulation will result in improved synthesis of bioactive compounds beneficial to human health, thus improving the quality of edible plants. Currently, we know little about the effect of PGRs on secondary-metabolite production in sweet potato. The present study aimed to investigate the effects of MeJA, ABA and SA on assessment of synthesis of phenolic and flavonoid compounds, anthocyanin and β-carotene in relation to PAL enzyme activity as well as the antioxidant and anticancer activities in sweet potato, Garnet yam cultivar.

## Methods

### Plant sampling and treatments

Seeds of the sweet potato cultivar Garnet Yam were sterilized with 1 % hypochlorite solution for 2–3 min, and then washed thoroughly with tap water. Clean seeds were sown in plastic boxes filled with compost. After 7 days, seedlings were transplanted into 10-L pots containing garden soil and sand (4:1 v/v), with two plants per pot. The plants were grown in a greenhouse at 25–31 °C and 70 % relative humidity, with a photosynthetic photon flux density (PPFD) of 600–800 μmol/m^2^/s at plant level. The plants were watered every two days (1.2 L).  Fertilizers (Nitrogen 100kg/ha; Phosphorus 90 kg/ha; Potassium 200 kg/ha and Calcium 200kg/ha) and all necessary micronutrients was applied in recommended dose. We selected three groups of equally sized, 21-day-old plants for treatment with 100 μM of MeJA, SA, and ABA. A control group of plants was also selected. Phytohormone concentrations (MeJA: 13 × 10^−6^ M; SA: 10^−5^ M; ABA: 10^−4^ M) were chosen based on previous screening experiments (data not published) demonstrating that these concentrations did not negatively affect plant health and growth. The PGRs were applied as a foliar spray once per week for 16 weeks. Control plants were irrigated with pure water. After 16 weeks, plants were harvested and immediately transported to the laboratory. Fresh samples (light-purple-fleshed root) were washed with pure water and were used to measure enzymatic activity. A second set of samples was frozen and lyophilized in a freeze dryer, then stored at −20 °C for further analyses. Seeds of sweet potato Garnet yam cultivar were donated from seed and plant certification institute (SPSRI, Tehran, Iran). Plant samples were submitted to SPSRI and were identified as sweet potato Garnet yam cultivar with voucher specimen of IK455/02. Voucher specimens deposited at herbarium of SPSRI.

### Extraction

Dried samples (5 g) were grounded into powder followed by extraction with distilled water (100 mL). Solutions were refluxed for 2 h at 65 °C, then cooled and filtered through Whatman filter paper (No. 1) in a filter funnel, followed by evaporation under reduced pressure in an Eyela rotary evaporator to remove excess solvent. Residue was freeze dried and dried extracts were kept at −20 °C for future analysis.

### Estimation of total phenolic contents (TPC)

Total phenolic content was determined by a spectrophotometric method according to Folin–Ciocalteu assay. Crude extracts (0.25 mg) was dissolved in methanol (10 mL) and 200 μL of solution were diluted in 20 mL of distilled water. Folin-Ciocalteu reagent (10-fold diluted; 1 mL) was added and the mixture was incubated in total darkness for 10 min at room temperature. After this time, sodium carbonate 7.5 % (1 mL) was added and incubated for 30 min, then the absorbance of the solution was read at 765 nm using a spectrophotometer (UV2550, Shimadzu, Japan). Different concentrations of gallic acid (0.062, 0.125, 0.250, 0.500 and 1 mg/mL) were used to prepare a calibration curve. Results were expressed as milligram gallic acid equivalents (GAE)/g dry matter (DM). Measurement was performed in triplicate and values are the average of three replicates.

### Estimation of total flavonoid contents (TFC)

Total flavonoid content of the crude extracts was determined by using the aluminium chloride spectrophotometric method. Crude extract (0.25 mg) was dissolved in methanol (10 mL). Extracts (1 mL) were mixed with NaNO_2_ solution (4 mL, 1:5, w/v) and incubated at room temperature for 6 min. After this time, 0.3 mL of AlCl_3_ solution (1:10, w/v) was added, the reagents were mixed well, and the reaction was allowed to stand for another 6 min. Immediately after that, 1 M NaOH solution (2.0 mL) was added to each extract and incubated for 10 min at room temperature. The absorbance of the solutions was read at 510 nm using a spectrophotometer (UV2550, Shimadzu, Japan). Different concentrations (0.031, 0.062, 0.125, 0.250, 0.500 mg/mL) of quercetin standard were used to prepare a calibration curve. Results were expressed as milligram quercetin equivalents (QE)/ g DM. Measurement was performed in triplicate and values are the average of three replicates.

### Estimation of total anthocyanin content (TAC)

Sweet potato root samples (50 mg) were extracted with methanol/HCl (99:1 v/v) solution at 4 °C for overnight. The observation of each sample were measured at 530 and 657 nm using a spectrophotometer (UV-2120 Optizen, Mecasys, Korea), and relative anthocyanin levels were determined with the equation OD530 − (0.25× OD657) × extraction volume (mL) × 1/weight of sample (g). Cyanidin 3-glucoside was used as a standard and results were expressed as miligrams of cyanidin 3-glucoside equivalents (Cy3-GE)/g DM.

### β-Carotene extraction and analysis

Freeze-dried sweet potato powder (5 g) was mixed with 2 g of calcium carbonate, 1 g of diatomaceous earth, and 25 ml of methanol. A hexane–acetone (1:1) mixture (50 ml) was added and stirred. The mixture was filtered under vacuum through a funnel with a fritted disk. The residue in the funnel was washed twice with 25 ml of methanol and then by 50 ml of the hexane– acetone mixture. All of the extracts were combined in a 250 ml separating funnel and washed with water. A few drops of saturated sodium chloride solution were added to the funnel to facilitate phase separation. The aqueous phase was discarded and the upper layer was transferred to a 50 ml volumetric flask and made to volume with hexane [3]. Samples were stored in dark vials at 20 °̊C until analysis.

The carotene content was analysed by a UHPLC system (1290 Infinity Quaternary LC System, Agilent, Santa Clara, CA, USA). The chromatographic system conditions were set as follows: mobile phase, methanol, acetonitrile and chloroform (42.5/42.5/15 v/v) HPLC grade; detector, UV 450 nm; column, C18 column (3.5 μm, 4.6 mm inner diameter [ID] × 100 mm); column oven temperature, 35 °C; injection volume 20 μl, and flow rate, 1.2 mL/min. Standard solutions of β-carotene with concentrations from 0.5 to 10 μg/mL were used to obtain a standard curve. Linear regression equations were calculated using Y = aX ± b, where X is the concentration of the related compound and Y the peak area of the compound obtained from UHPLC. The linearity was established by the coefficient of determination (R^2^). UHPLC analysis was performed in triplicate and values are the average of three replicates.

### Separation and analysis of flavonoids and phenolic acids

Ultra-high performance liquid chromatography (UHPLC, 1290 Infinity Quaternary LC System, Agilent, Santa Clara, CA, USA) was used to separate and identify the phenolic acids. The chromatographic system conditions were set as follows: mobile phase, 0.03 M orthophosphoric acid (A) and methanol HPLC grade (B); detector, UV 280-360 nm; column, C18 column (5.0 μm, 4.6 mm inner diameter [ID] × 250 mm); column oven temperature, 35 °C; and flow rate, 1.0 mL/min. Gradient elution was performed as follows: 0–10 min, 10 % B; 10–15 min, 50 % B; 15–20 min, 100 % B; and finally 5 min for washing. All standards (quercetin, luteolin, myricetin, kaempferol, gallic acid, chlorogenic acid, 3,5-Di-caffeoylquinic acid and 4,5-Di-caffeoylquinic acid were purchased from Sigma-Aldrich (M) Sdn Bhd, Selangor, Malaysia) were dissolved in methanol HPLC grade. Linear regression equations were calculated using Y = aX ± b, where X is the concentration of the related compound and Y the peak area of the compound obtained from UHPLC. The linearity was established by the coefficient of determination (R^2^). UHPLC analysis was performed in triplicate and values are the average of three replicates.

### PAL extraction and assay

Sweet potato was ground with pestle and mortar and was well mixed with acetone, placed in a freezer for 15 min, and then centrifuged at 20,000 × g at 4̊C for 15 min. The pellet was dried under vacuum and extracted at 4̊C by gentle stirring with 100 mM sodium borate buffer (5 ml/g ), pH 8.8, containing 5 mM ß-mercaptoethanol, 2 mM EDTA. After 1 h, the solution was filtered through one layer of nylon cloth and centrifuged at 20,000 × g at 4̊C for 15 min. PAL activity in the buffer supernatant was determined by the production of cinnamate during 1 h at 30̊C, as measured by the absorbance change at 290 nm. The assay mixture contained 15 mol L-phenylalanine, 30 mM sodium borate buffer (pH 8.8), and 0.2 to 0.5 ml buffer supernatant, depending on the PAL activity level, in a total volume of 3.0 ml. The substrate was added after 10 min of pre incubation and the reactions stopped with 0.1 ml 6 N HCl. Assays were performed in triplicate [28].

### In vitro evaluation of antioxidant activity

#### 1,1-Diphenyl-2-picrylhydrazyl (DPPH) assay

The DPPH assay was used in order to evaluate the free radical scavenging activity of sweet potato extracts. DPPH was dissolved in methanol at a concentration of 100 μM. The DPPH solution (3 mL) was mixed with 3 mL of various concentrations of plant extracts and incubated in a dark room for 20 min at 27 °C. After incubation, the absorbance of the samples was read at 517 nm using a spectrophotometer (UV2550, Shimadzu, Japan). α-tocopherol was used as a positive control. The scavenging activity was calculated using the following formula:

$$ \%\ \mathrm{inhibition} = \kern0.5em \left[\left(\mathrm{absorbanc}{\mathrm{e}}_{\mathrm{control}}\hbox{--}\ \mathrm{absorbanc}{\mathrm{e}}_{\mathrm{sample}}\right)/\mathrm{absorbanc}{\mathrm{e}}_{\mathrm{control}}\right)\Big] \times 100 $$

#### β-carotene bleaching assay

β-carotene (2 mg) was dissolved in 10 ml of chloroform linoleic acid (40 mg) and Tween 40 (400 mg) were added to the flask with vigorous shaking. The chloroform was removed at 40 °̊C under vacuum and 100 mL of distilled water was then added to the residue. Aliquots (4.8 ml) of this emulsion were transferred into different test tubes containing 0.2 ml of different concentrations of the sweet potato extracts. The tubes were shaken and incubated at 50 ˚C in a water bath. As soon as the emulsion was added to each tube, the zero time absorbance was measured at 470 nm using a spectrophotometer. Absorbance readings were then recorded at 20 min intervals until the control sample had changed colour (for 2h). A blank, devoid of β -carotene, was prepared for background subtraction. Antioxidant activity was calculated using the following equation:

Antioxidant activity = (β -carotene content after 2 h of assay/initial β -carotene content) × 100. Where β -carotene content after 2 h of assay is : (the absorbance of the antioxidant at 2h - absorbance of the control at 2h), and initial β -carotene content is: (the absorbance of the control at 0 h - absorbance of the control at 2h). The assays were carried out in triplicate and the results expressed as mean values ± standard deviations.

### Determination of anticancer activity

#### Cell culture and treatment

Estrogen receptor (ER)-positive MCF-7, ER-negative MDA-MB-231 breast cancer cells and normal cell line (MCF-10A) were procured from the laboratory of Molecular Biomedicie, Institute Bio-sience, Universiti Putra Malaysia, Serdang, Selangor, Malaysia. Cells were cultured in RPMI 1640 media containing 10 % fetal bovine serum (FBS). Cell lines were incubated overnight at 37 °C in 5 % CO_2_ for cell attachment. The cells were maintained by sub-culturing in 25 cm^2^ tissue culture flasks. Cells growing in the exponential phase were used for cell viability assay.

#### MTT (3-(4,5-Dimethylthiazol-2-yl)-2,5-diphenyltetrazolium bromide) assay

The assay was conducted as follows: Cancer cells were seeded in 96-well plates at a density of 1 × 10^4^ cells/well in 100 μL of media. After 24 h, the medium was removed and the cells were incubated for 3 days in the presence and absence of various concentrations of sweet potato root extract [test extracts were prepared in 0.1 % Dimethyl sulfoxide (DMSO) and serially diluted with media to obtain appropriate concentrations]. The following concentrations of extracts were used: 0.25, 0.50, 1, 2 and 4 mg/mL. Cells in the control group received only media containing 0.1 % DMSO. After incubation, the test compound containing media was removed and washed with 200 μL of PBS followed by addition of 20 μL of MTT reagent (5 mg/mL MTT in PBS) and incubated for 4 h at 37 °C. The medium was removed and 100 μL DMSO was added and the absorbance measured using a micro plate reader at 540 nm followed by the calculation of percentage viability. 0.1 % (v/v) DMSO in medium was used as negative control. The cell viability was determined using the formula:

$$ \mathrm{Cell}\ \mathrm{viability}\ \left(\%\right) = 100\kern0.5em \hbox{--} \kern0.5em \left(\left[\mathrm{X}-\mathrm{Y}\right]/\mathrm{X}\right) \times 100 $$

where X = absorbance of cells treated with 0.1% DMSO medium, Y = absorbance of cells treated with extracts.

Each point represents the mean of triplicate experiments.

### Statistical analysis

Statistical analysis was performed using Statistical Analysis System (SAS version 9.2, SAS Institute Inc.). Mean separation test between treatments was performed using Duncanʼs Multiple Range Test and P-value of < 0.05 was regarded as significant.

## Results and discussion

### Effect of PGR treatments on total phenolic content (TPC), total flavonoid content (TFC), total anthocyanin content (TAC) and β-carotene content

The results of present study showed that PGR treatments induced the accumulation of TPC, TFC, TAC, and β-carotene in sweet potato root (Table 1). MeJA resulted in the highest TPC (9.42 mg GAE/g DM), followed by SA (8.26 mg GAE/g DM) and ABA (7.60 mg GAE/g DM); the differences were significant compared with those in the control plants (5.74 mg GAE/g DM). Similarly, PGR application led to significantly higher TFC than in the control (1.78 mg QE/g DM). As with the phenolic acids, MeJA treatment yielded the highest TFC (5.96 mg QE/g DM), followed by SA (5.17 mg QE/g DM) and ABA (4.88 mg QE/g DM). Our data demonstrated that total phenolic and flavonoid contents rose in PGR-treated plants. MeJA and SA treatments have been previously reported to increase total phenolic content in *Brassica alboglabra* [29] and *Lactuca sativa* [30]. Consistent with our results, total phenolic and flavonoid contents were increased by MeJA, SA, and ABA in *Panax ginseng* [31], *Ocimum basilicum* [25] and *Lactuca sativa* [19] respectively.

**Table 1:** Effects of MeJA, SA and ABA on assessment of total phenolic content, total flavonoid content, total anthocyanin content and β-carotene content in sweet potato root

Treatments	TPC (mg GAE /g DM)	TFC (mg QE /g DM)	TAC (mg Cy3GE/g DM)	β-carotene (mg/g DM)
Control	5.74 ± 0.35^d^	1.78 ± 0.02^d^	0.20 ± 0.01^d^	20.90 ± 2.11^c^
MeJA	9.42 ± 0.33^a^	5.96 ± 0.31^a^	0.66 ± 0.04^a^	56.77 ± 3.56^a^
SA	8.26 ± 0.31^b^	5.17 ± 0.22^b^	0.28 ± 0.03^c^	41.29 ± 2.88^b^
ABA	7.60 ± 0.27^c^	4.88 ± 0.02^c^	0.52 ± 0.02^b^	40.11 ± 3.15^b^

Data are means of triplicate measurements ± standard deviation. Means not sharing a common single letter in each column for each measurement were significantly different at *P* < 0.05

Significant differences between treatments were observed for TAC. MeJA-treatment resulted in the highest TAC (0.66 mg Cy3GE/g DM), followed by ABA (0.52 mg Cy3GE/g DM), then SA (0.28 mg Cy3GE/g DM) compared to levels in control plants (0.2 mg Cy3GE/g DM; Table 1). Anthocyanins are pigment-containing compounds responsible for colors such as purple, red, or black in plants; in sweet potatoes, they are noted for their stability and physiological function [32]. Consistent with this observation, anthocyanin content in MeJA-treated Arabidopsis [33] and *Kalanchoe blossfeldiana* [34] seedlings were greater than in control. Similarly, SA induced anthocyanin [16], flavonoids and phenolics [35] synthesis in *Zingiber officinale*, and total anthocyanin content in red lettuce treated with ABA was significantly higher than in controls [19]. In sweet potatoes, jasmonic-acid treatment changed the root color to red, while control roots maintained the original white color [26]. Our study is consistent with these previous outcomes, and together the data suggest jasmonate involvement in the anthocyanin metabolic pathway of sweet potato. The presence of MeJA might trigger the expression of anthocyanin-synthesis genes in non-pigmented cells, causing an increase in total pigment accumulation.

Sweet potato root treated with MeJA exhibited the highest beta-carotene content (56.77 mg/g DM) followed by SA (41.29 mg/g DM) and ABA (40.11 mg/g DM) compared to control plants (20.90 mg/g DM); the differences were all significant compared with control. Barickman et al. [36] reported that higher ABA concentrations caused significant increases in β-carotene, lutein, and neoxanthin in tomato leaf tissue. Plant color and variety can influence the concentrations and profiles of phenolics, as well as of anthocyanins [37] and carotenoids [7]. For example, the roots of purple sweet potato cultivars have high anthocyanin content, and these anthocyanins are also more stable than those of other plants that are purple-red in color [38].

### Effect of PGR treatments on individual phenolic and flavonoid compounds

Table 2 shows the results of UHPLC analyses on phenolics and flavonoids in treated- and control-plant extracts. We identified four flavonoids and four phenolic acids from sweet potato roots. Flavonoids and phenolics synthesis were induced by PGR application on sweet potato. With the exception of luteolin, MeJA treatment resulted in the highest flavonoid and phenolic content. Specifically, quercetin content was highest under MeJA treatment (1.16 mg/g DM), followed by ABA (0.95 mg/g DM) and SA (0.83 mg/g DM) as against levels in control plants (0.32 mg/g DM). Kaempferol content was highest under MeJA treatment (0.93 mg/g DM), followed by ABA (0.82 mg/g DM) and SA (0.79 mg/g DM) as against levels in control plants (0.28 mg/g DM). Myricetin was higher in MeJA treatments (0.98 mg/g DM), followed by SA (0.86 mg/g DM) and ABA (0.77 mg/g DM); all were significantly higher than control (0.34 mg/g DM). Sweet potato treated with SA exhibited highest content of luteolin (0.84 mg/g DM) followed by MeJA (0.75 mg/g DM) and ABA (0.61 mg/g DM) compared to control plants (0.22 mg/g DM).

**Table 2:** Identification of flavonoids and phenolic acids compounds from sweet potato root extract treated by MeJA, SA and ABA

Compounds	Control	MeJA	SA	ABA
Quercetin	0.32 ± 0.08^d^	1.16 ± 0.14^a^	0.83 ± 0.04^c^	0.95 ± 0.05^b^
Kaempferol	0.28 ± 0.08^c^	0.93 ± 0.09^a^	0.79 ± 0.05^b^	0.82 ± 0.07^b^
Myricetin	0.34 ± 0.02^d^	0.98 ± 0.09^a^	0.86 ± 0.05^b^	0.77 ± 0.03^c^
Luteolin	0.22 ± 0.06^d^	0.75 ± 0.05^b^	0.84 ± 0.07^a^	0.61 ± 0.04^c^
Gallic acid	0.31 ± 0.07^c^	0.81 ± 0.08^a^	0.46 ± 0.07^b^	0.40 ± 0.06^b^
Chlorogenic acid	0.52 ± 0.04^c^	0.98 ± 0.14^a^	0.61 ± 0.05^b^	0.55 ± 0.03^c^
3,5-dicaffeoylquinic acid	0.38 ± 0.05^d^	0.75 ± 0.08^a^	0.59 ± 0.05^b^	0.49 ± 0.04^c^
4,5-dicaffeoylquinic acid	0.50 ± 0.02^c^	0.89 ± 0.10^a^	0.65 ± 0.06^b^	0.60 ± 0.08^b^

Data are means of triplicate measurements ± standard deviation. Means not sharing a common single letter in each row for each measurement were significantly different at *P* < 0.05

Sweet potato treated with MeJA exhibited the highest concentrations of gallic acid, chlorogenic acid, 3,5- dicaffeoylquinic acid, and 4,5- dicaffeoylquinic acid (0.81, 0.98, 0.75, and 0.89 mg/g DM, respectively), followed by SA (0.46, 0.61, 0.59, and 0.65 mg/g DM) and ABA (0.40, 0.55, 0.49, and 0.60 mg/g DM) treatments. These values were significantly higher than those of the control. Quercetin and chlorogenic acid were identified as abundant flavonoid and phenolic compound respectively in sweet potato.

Oki et al. [39] identified the following phenolic compounds in sweet potatoes: chlorogenic acid, isochlorogenic acid, cinnamic acid, anthocyanins, cyanidin, and peonidin aglycones Polyphenols are bioactive molecules universally distributed in different plant species that play important roles in plant responses to biotic and abiotic stresses and can be induced by environmental stresses and elicitors [40]. Several phenylpropanoid derivatives play a role in plant defense against biotic and abiotic stresses [41].

**Fig. 1:**
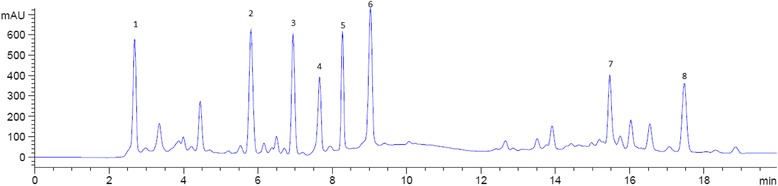
Full chromatogram of UHPLC analysis of sweet potato root extract (treated with MeJA) showing peak corresponding to gallic acid (1), chlorogenic acid (2), myricetin (3), luteolin (4), kaempferol (5), quercetin (6), 3,5-dicaffeoylquinic acid (7) and 4,5-dicaffeoylquinic acid (8)

SA treatment enhances rutin accumulation in India buckwheat (*Fagopyrum tartaricum)* [42] and MeJA treatment induces myricetin, quercetin, and kaempferol in red raspberries (*Rubus idaeus* L.) [43] and kale (*Brassica oleracea *L.) leaves [44]. Consistent with our current study, chlorogenic acid was lower in MeJA-treated lettuce than in the control [30]. Notably, PGRs appeared to exert a stronger effect on flavonoid synthesis than on phenol synthesis. Figure 1 show full chromatogram of UHPLC analysis of sweet potato root extracts (treated with MeJA).

### Effect of PGR treatments on PAL activity

**Fig. 2:**
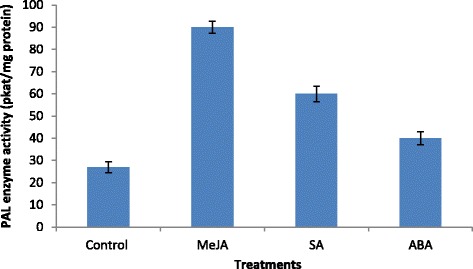
Effects of MeJA, SA and ABA on PAL activity in sweet potato root extracts

PAL plays a crucial role at the interface between plant primary and secondary metabolism by catalysing the deamination of l-phenylalanine to form trans-cinnamic acid, the first step in the general phenylpropanoid way. Thousands of secondary metabolic products in plants such as flavonoids, anthocyanins, lignins, and phytoalexins are derived from phenylpropanoid [23]. Treatment with PGRs significantly influenced PAL activity in sweet potato (*P* < 0.05), (Fig. 2). PAL activity was highest in plants treated with MeJA (87.0 pkat/mg protein), followed by that in plants treated with SA (64.0 pkat/mg protein) and ABA (49.0 pkat/mg protein) and that in control plants (27.0 pkat/mg protein). Hung and Kao [45] reported that ABA increases the activity of PAL, a key regulatory enzyme of anthocyanin biosynthesis, and thereby promotes senescence of rice leaves. Moreover, gibberellic acid enhanced PAL activity in young plants of *Fragaria ananassa* ‘Chandler’ [46]. Here, PGR application greatly induced PAL activity and thus enhanced the production of phenolic acids, flavonoids, and anthocyanins. When PAL activity was low (in control plants), flavonoid content was also low and vice versa. Thus, our data suggest that under normal conditions, post-PAL intermediates are unavailable for use in flavonoid synthesis, and high PAL activity is required for flavonoid accumulation. This conclusion is consistent with the commonly held view that PAL is the main limiting factor in the biosynthesis of flavonoids, cinnamic acids, and other phenylpropanoids [22, 24]. Because the enzyme is likely key in channeling phenylalanine to phenolic synthesis, and hence to flavonoid synthesis.

### Effect of PGR treatments on antioxidant activity

**Fig. 3:**
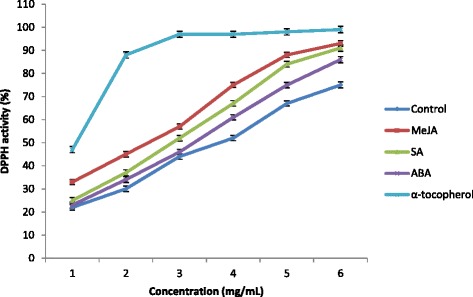
Effects of MeJA, SA and ABA on antioxidant activity of sweet potato root using DPPH assay

The antioxidant capacity of treated and control plants were determined using a DPPH free-radical scavenging assay and a β-carotene bleaching assay. The former assay measures the hydrogen-donating ability of antioxidants, and activity is calculated as the relative decrease in DPPH absorbance as it reacts with the antioxidant [47]. As can be seen from the Fig. 3, DPPH radical scavenging activity of sweet potato was increased significantly (*P* < 0.05) as sample concentration increased. Furthermore, treatment with PGRs influenced free-radical scavenging activity—the highest scavenging activity was observed in plants treated with MeJA, followed by plants treated with SA and ABA, and was lowest in the control plants (Fig. 3). At a sample concentration of 5 mg/mL, MeJA-treated plants exhibited the highest DPPH activity (88.21 %), followed by SA (84.11 %), ABA (75.32 %), and control plants (67.84 %). The half-maximal inhibitory concentration (IC_50_) values across the four conditions were, respectively, 2.40, 3.0, 3.40, and 4.20 mg/mL, as compared with that of α-tocopherol (98.12 %; 1.1 mg/mL)., Table 3). Our results are comparable to the range of IC_50_ values (0.49–5.23 mg/mL) obtained by Huang et al. [48], using the same method on various sweet potato genotypes. The IC_50_ for all PGR treatments were significantly different (*P* < 0.05) in terms of radical scavenging activity. Lower IC_50_ values represent stronger free radical inhibition—strong free-radical inhibitors are active at low concentrations.

**Table 3:** IC_50_ value (mg/mL) of antioxidant activity of sweet potato extract

	DPPH asssay	β-carotene bleaching assay
Control	4.2 ± 0.11^a^	4.5 ± 0.25^a^
MeJA	2.4 ± 0.20^d^	2.9 ± 0.15^d^
SA	3.0 ± 0.27^c^	3.3 ± 0.21^c^
ABA	3.4 ± 0.25^b^	3.7 ± 0.22^b^
α-tocopherol	1.1 ± 0.18^e^	1.3 ± 0.12^e^

Data are means of triplicate measurements ± standard deviation. Means not sharing a common single letter in each column for each measurement were significantly different at *P* < 0.05

**Fig. 4:**
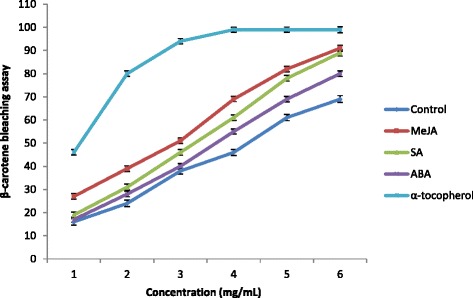
Effects of MeJA, SA and ABA on antioxidant activity of sweet potato root using β-carotene bleaching assay

Several studies have reported on the antioxidant activity of sweet potato extracts [3, 48, 49], which is mainly ascribed to the concentration of phenolic and flavonoid compounds in the plants [50, 51]. In this study, the antioxidant activity of sweet potato extracts treated with different PGRs was evaluated using the β-carotene-linoleate bleaching method because β-carotene was detected from extracts. Moreover, based on previous studies, β-carotene shows strong biological activity and constitutes a physiologically important compound [52, 53]. Antioxidant activity is measured through the loss of the yellow color from β-carotene as it reacts with radicals formed from linoleic acid oxidation in emulsion. The antioxidant activity of sweet potato increased significantly (*P* < 0.05) as sample concentration increased (Fig. 4). Furthermore, treatment with PGRs significantly influenced free-radical scavenging activity; all extracts had lower antioxidant activities than α-tocopherol. MeJA-treated plants showed the highest β-carotene blenching activity (with an IC_50_ value of 2.90 mg/mL), followed by SA (3.30 mg/mL) and ABA (3.70 mg/mL) as against that of the control plants (4.5 mg/mL), (Table 3). In summary, antioxidant activity was high in the PGR-treated plants, corroborating previous research demonstrating that MeJA and SA treatment significantly increased antioxidant activity in *Brassica alboglabra* [29]. Agarwal et al. [54] determined that the exogenous effect of SA and ABA increased the activity of the antioxidant enzymes of wheat genotypes. Finally, results from our previous studies have revealed that foliar application of SA enhanced antioxidant activity in ginger varieties via inducing antioxidant enzymes activities [55] and phytochemical production [16, 17].

### Anticancer activity

**Fig. 5:**
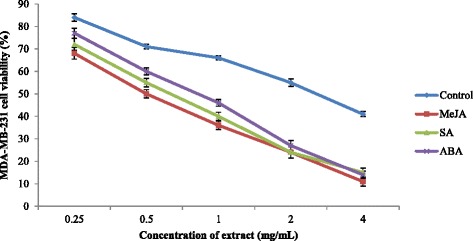
Anticancer activity of sweet potato root extracts treated with MeJA, SA and ABA against MDA-MB-231 breast cancer cell

**Fig. 6:**
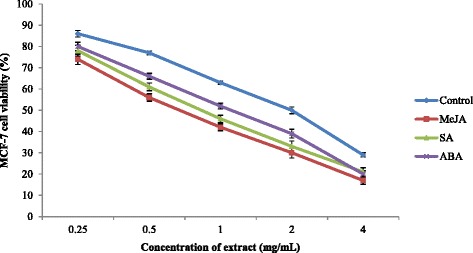
Anticancer activity of sweet potato root extracts treated with MeJA, SA and ABA against MCF-7 breast cancer cell

**Fig. 7:**
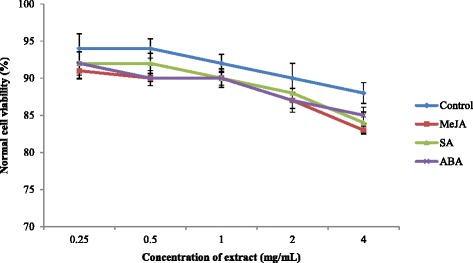
Toxicity effect of sweet potato root extracts treated with MeJA, SA and ABA

Root extracts of sweet potato (0.25-4 mg/mL) were tested for anticancer activity against the breast cancer cell lines MCF-7 and MDA-MB-231 (Figs. 5 and 6). Significant differences (*P* < 0.05) between the IC_50_ values of sweet potato extracts treated with different PGRs were observed. Extracts of sweet potato treated with MeJA exhibited more potent anticancer activity against MDA-MB-231 and MCF-7 cell lines with IC_50_ value of 0.66 and 0.62 mg/mL, respectively followed by SA (0.78 and 0.90 mg/mL) and ABA (0.94 and 1.40 mg/mL) treated plants (Table 4). The IC_50_ values of all extracts were higher than that of Tamoxifen (MDA-MB-231 = 0.038 mg/mL; MCF-7 = 0.038 mg/mL), an anticancer drug. Extracts treated with PGRs at concentrations of 0.25–4 mg/mL were not toxic to a normal cell line, which exhibited a viability range of 83–92 % (Fig. 7). In an herbal supplement, one ingredient may provide the desired therapeutic benefits while others may have toxic effects for humans. For example, many Malaysian herbs and spices may also contain toxic components that have been poorly investigated. In this study, we evaluated sweet-potato-root extracts and found that they were nontoxic for normal (MCF-10A) cells. The ~80–90 % cell viability falls well above the range recommended by the American National Cancer Institute, which considers crude herbal extracts that do not decrease the viability of normal cells below 76 % as safe for human consumption [56]. Additionally, our findings in this study are in accordance with research by Sugata et al. [57] who showed that extracts of purple-fleshed sweet potatoes inhibited 50 % of the MCF-7 cancer cell line at concentrations of 3.4–5.9 mg/mL.

**Table 4:** IC_50_ value (mg/mL) of anticancer activity of sweet potato extract

	MDA-MB-231	MCF-7
Control	3.40 ± 0.314^a^	2.68 ± 0.221^a^
MeJA	0.66 ± 0.095^d^	0.62 ± 0.150^d^
SA	0.78 ± 0.103^c^	0.90 ± 0.131^c^
ABA	0.94 ± 0.161^b^	1.40 ± 0.264^b^
Tamoxifen	0.038 ± 0.007^e^	0.037 ± 0.004^e^

Data are means of triplicate measurements ± standard deviation. Means not sharing a common single letter in each column for each measurement were significantly different at *P* < 0.05

Previous reports have suggested that sweet potato extracts exert significant antiproliferative and antimetastatic effects on human colorectal cancer cell lines, both in vitro and in vivo [58]. Moreover, the constituent polyphenolics of sweet potato greens have displayed antimutagenic, antidiabetic, antibacterial, anti-inflammatory, and anticancer activity [4]. We now know that the anticancer properties of herbs and spices are directly related to their phytochemical content [59]. As we have described, sweet potato roots with the highest concentrations of secondary metabolites (e.g., anthocyanin, β-carotene, phenolics, and flavonoids) exhibited the most potent antioxidant and anticancer activity, strongly suggesting that the potent antioxidant and anticancer activity observed in MeJA-treated sweet potatoes can be attributed to the phytohormone inducing high phytochemical content. Although more research is needed to better understand the association between sweet-potato phytochemicals and anticancer activity, this study corroborates the findings of a great deal of the previous work on this topic.

### Correlation analysis

It is important to examine correlation between phytochemical content and biological activity of crops and herbs in order to introduce corresponding compound for biological activity in each plants. Knowing of this could help researcher to establish suitable condition or to use suitable techniques in order to enhance this highlighted compounds. In current study, correlation coefficient analyses showed a significant relationship between phytochemical content and breast cancers proliferation. A negative and significant correlation coefficient was also found between breast cancers proliferation and activities of antioxidants ranging from R^2^ = −0.7947 to −0.9100 (Table 5). Significant correlation between antioxidant activity and breast cancer cell line (MCF-7) proliferation of the five herbal extracts was reported previously [60]. Between identified phytochemicals, TAC and TFC showed a strong negative correlation with MCF-7 (R^2^_TAC_ = −0.9226; R^2^_TFC_ = −0.9128) and MDA-MB-231 (R^2^_TAC_ = −0.9371; R^2^_TFC_ = −0.9233) cancer proliferation. In current study, correlation between identified phytochemicals and MCF-7 cancer proliferation decreased in the following order: TAC > myrecitin > TFC > kaempferol > TPC > gallic acid > quercetin > 4,5-dicaffeoylquinic acid > chlorogenic acid > luteolin > 3,5-dicaffeoylquinic acid. Also, correlation between identified phytochemicals and MDA-MB-231 cancer proliferation decreased in the following order: TAC > TFC > gallic acid > kaempferol > TPC > quercetin > 4,5-dicaffeoylquinic acid > chlorogenic acid > myrecitin >3,5-dicaffeoylquinic acid > luteolin.

**Table 5:** Correlations (R^2^) among human breast cancer cell proliferation, and identified phytochemicals from root extracts of sweet potato

	MCF-7	MDA-MB-231
TFC	−0.9128^**^	−0.9233^**^
TPC	−0.8872^**^	−0.9024^**^
quercetin	−0.8563^**^	−0.8931^**^
kaempferol	−0.8927^**^	−0.9077^**^
myrecitin	−0.9162^**^	−0.8255^**^
luteolin	−0.7311^*^	−0.7174^*^
gallic acid	−0.8841^**^	−0.9129^**^
chlorogenic acid	−0.7499^*^	−0.8335^**^
4,5-dicaffeoylquinic acid	−0.7626^*^	−0.8825^**^
3,5-dicaffeoylquinic acid	−0.7129^*^	−0.8104^**^
TAC	−0.9226^**^	−0.9371^**^
β-carotene	−0.8744^**^	−0.8969^**^
DPPH	−0.9100^**^	−0.7947^**^
β-carotene blenching assay	−0.8864^**^	−0.8812^**^

^*^ and ^**^ are significant at *P* < 0.05 and 0.01 respectively

Several studies reported a significant correlation between the pharmaceutical activity (antioxidant and anticancer activities) of herbs and the phytochemical content [27, 59, 61]. Based on correlation analysis it seems that anthocyanins and flavonoids are corresponding compounds in sweet potato root extracts for anticancer activity against breast cancer cell lines.

## Conclusion

This study clearly demonstrated that the sweet potato is a suitable source of natural bioactive compounds, possessing antioxidant and anticancer activity that could be attractive to the food or pharmaceutical industries. We evaluated whether PGR treatments (MeJA, ABA, and SA) could enhance the content of health-promoting phytochemicals in sweet potato root. We found that compared with the other two PGR treatments, MeJA-treated plants possessed higher concentrations of total phenolics, flavonoids, anthocyanins, and β-carotene, as well as higher amounts of individual phenolics and flavonoids. The antioxidant activity of sweet potato root extracts improved significantly over that of untreated plants with foliar application of MeJA, with less improvement seen following treatment with SA and ABA. Furthermore, the data presented here on the composition of bioactive compounds and related antioxidant activity should prove useful in standardizing sweet potato extracts for pharmaceutical applications. Additionally, a major finding to emerge from this study is that the sweet-potato-root extracts exhibited a promising anticancer activity against the MDA-MB-231 and MCF-7 breast-cancer cell lines. The extracts contained substantial amounts of flavonoid compounds, such as myricetin, quercetin, and anthocyanin, all of which potently inhibited the growth of breast cancer cells. Subsequently, our MTT assay indicated that MeJA-treated sweet potatoes are a potential source of anticarcinogenic compounds. In closing, we observed strong potential for the enhanced production of health-promoting phytochemicals using PGRs such as MeJA, which has important implications for improving the health benefits of sweet potato-based functional food.

## Abbreviations

ABA: abscisic acid
DMSO: dimethyl sulfoxide
DPPH: 1,1-diphenyl-2-picrylhydrazyl
FBS: fetal bovine serum
IC_50_: half-maximal inhibitory concentration
MeJa: methyl jasmonate
MTT: (3-(4,5-Dimethylthiazol-2-yl)-2,5-diphenyltetrazolium bromide)
PAL: phenylalanine ammonia-lyase
SA: salicylic acid
TAC: total anthocyanin content
TCC: total β-carotene content
TFC: total flavonoid content
TPC: total phenolic content
UHPLC: ultra-high performance liquid chromatography
